# Phytochemical analysis and docking study of compounds present in a polyherbal preparation used in the treatment of dermatophytosis

**DOI:** 10.29252/cmm.3.4.6

**Published:** 2017-12

**Authors:** Nagesh Simhadri VSDNA, Muthuchamy Muniappan, Iyanar Kannan, Subramanyam Viswanathan

**Affiliations:** 1 Department of Pharmacology, Tagore Medical College and Hospital, Chennai, India; 2 Department of Pharmacology, Sree Balaji Medical College and Hospital, Chennai, India; 3 Department of Microbiology, Tagore Medical College and Hospital, Chennai, India; 4 Department of Pharmacology, Meenakshi Medical College and Hospital, Chennai, India

**Keywords:** Anti-fungal activity, Chitinase, Dermatophytes, Fungalysin, Lumazine synthase, Molecular docking, Soleshine, 1, 3-β-Glucan synthase

## Abstract

**Background and Purpose:**

Soleshine is a polyherbal preparation established in the market for the treatment of cracks and tinea pedis, which is applied externally. This preparation is composed of the extracts of indigenous plants, namely* Azadirachta indica, Lawsonia alba, *and *Shorea robusta*, mixed with castor oil and sesame oil. In the present study, an attempt was made to identify the constituents of soleshine and identify some potential drug-like molecules that can inhibit important drug targets of the dermatophytes using molecular docking method.

**Materials and Methods:**

The active ingredients of polyherbal preparation were identified with the aid of gas chromatography-mass spectrometry (GC-MS). Two major compounds were selected based on the retention time and percentage of the area covered in the graph for docking study. The three-dimensional structures of 1,3-β-glucan synthase, chitinase, fungalysin, and lumazine synthase were derived by homology modelling using MODELLER software, version 9.0. The docking of the ligand and receptor was performed using iGEMDOCK and AutodockVina software. The physicochemical properties, lipophilicity, hydrophilicity, and drug likeness properties were obtained from the Swiss ADME online server tool.

**Results:**

The GC-MS analysis demonstrated the presence of different phytochemical compounds in the extract of polyherbal preparation. A total of 20 compounds were identified, among which 3,7-dimethyl-2,6-octadienaland 2-pentene-2-methyl were the major compounds. Regarding 3,7-dimethyl-2,6-octadienal, the covered area and height were 40.15% and 46.17%, respectively. These values were 31.90% and 23.33% for 2-pentene-2-methyl, respectively. These two major compounds had an excellent binding affinity and obeyed the rules for the drug likeness and lead likeness.

**Conclusion:**

As the findings indicated, the two major ingredients present in soleshine showed a good antifungal activity as they inhibited the enzymes responsible for the survival of fungal organism; furthermore, they were appropriate for the lead molecules.

## Introduction

Diseases caused by fungi mark a vital threat to the health care and are among the critical causes of morbidity and mortality worldwide [1]. In the past, fungi were not considered as important pathogenic organisms as the annual mortality rate due to candidiasis was steady from 1950 to 1970 [2, 3]. However, from 1970 onwards, a significant increase was observed in death rate because of the indiscriminate use of immunosuppressant, broad-spectrum antimicrobial drugs, indwelling intravenous devices, and emergence of viral infections, such as AIDS. The dreadful consequences of fungal infections has necessitated the search for newer, safer, and more potent drugs [4]. 

Fungal cell wall is composed of chitin interlinked with 1,3-β-glucan, constituting 30-80% of the cell wall. 1, 3-β-Glucan synthase (EC 2.4.1.34) is an enzyme, which affects the synthesis of fungal cell wall. Chitin, a homopolymer of insoluble linear β 1,4-linked N-acetylglucosamine [5, 6], is a fibrous cellulose-like material, containing chitinases, a poly (1,4-β-[2-acetamido-2-deoxy-D-glucoside]) glycanohydrolases (EC 3.2.1.14), which are required for the synthesis of the cell wall [7]. Fungalysin, a metallopeptidase, cleaves the proteins and produces metabolic products to enable the activity of exoproteases, resulting in the provision of short peptides and free amino acids for the sustenance of fungal organisms [8]. 

Lumazine synthase (EC 2.5.1.78) is an enzyme involved in riboflavin (Vitamin B2) biosynthesis. Bacteria and fungi are not able to incorporate riboflavin exogenously. They absolutely rely on endogenous biosynthesis. Lumazine synthase catalyzes the important steps in riboflavin biosynthesis pathway [9, 10]. All these enzymes are excellent drug targets to prepare new anti-fungal medications.

Soleshine is a polyherbal preparation containing the extracts of the leaves of neem (*Azadirachta indica*) and henna (*Lawsonia alba*), resin of Sal tree (*Shorea robusta*), Sesame oil, and Castor oil. Neem belongs to Meliaceae family, a well-known plant with medicinal properties since olden days. All parts of *Azadirachta indica* have a myriad of medicinal properties [11, 12]. The leaf and bark of the neem plant is used in the treatment of gingivitis, periodontitis, sores, boils, enlarged spleen, malarial fever, fever during childbirth, measles, smallpox, head scald, and cutaneous affections. Neem oil is used as a contraceptive (through intravaginal route for the treatment of vaginal infections), insecticidal agent [13], and mosquito repellent. This oil contains crystalline compounds, namely nimbin and nimbinin, as well as amorphous bitter substance called nimbidin [14]. 

Henna belongs to the family of Lythraceae, the leaves of which are used in various ailments, such as disarray, jaundice, bleeding disorders, ulcers, prurigo skin diseases, giddiness, and vertigo [15]. The leaves contain naphthoquinones, particularly lawsone, coumarins (laxanthone, I, II, and III), flavonoids, luteolin and its 7-O-glucoside, acacetin-7-O-glucoside, and beta-sitosterol-3-O-glucoside. All parts of the plant contain tannins [16, 17]. The chloroform and ethanolic extracts of henna leaves exhibit promising anti-microbial activity against *Shigella *and *Vibrio cholera.*

Henna plant is used as a prophylactic medicament for the infection of hands and feet against mycosis. The antifungal activity is due to the presence of lawsone, a naphthoquinone that is a known secondary metabolite of henna. The extract of ethanol-water (1:1) of the bark exhibits hepatoprotective activity on carbonate-trachloride-induced liver toxicity. In some experiments with isoplumbagin and lawsaritol, the secondary metabolites obtained from the plant parts showed anti-inflammatory activity. 

*Shorea robusta,* known as a sal tree belongs to the family of Dipterocarpaceae. The bark, young leaves, twigs/leaves, and powder dust of this plant contain 7-12%, 20%, 22%, and 12% tannins, respectively. The aqueous extract of the bark of sal tree contains 39.6% of tannins with a trans/non-trans ratio of 0.73. Oleanolic acid has also been extracted from the bark. Moreover, several triterpenoids have been isolated from the sal resin [18, 19]. Hydroxyhopanone, dammarenediol II, and dammarenolic acid are reported to be effective as antiviral agents against *Herpes simplex. *The sal resin on dry distillation gives an essential oil, known as Chuaa oil containing 96.0% neutral, 3.0% phenolic fraction, and 1.9% acidic fractions. The non-phenolic portion of this oil has an anti-depressant effect on the central nervous system; however, the phenolic portion is less effective. 

Sesame oil is obtained from the seeds of *Sesamum indicum* (Family: Pedaliaceae). The oil obtained from sesame seeds is higher in content (around 50%) than that obtained from other seeds [20]. The sesame seeds contain 40-60% oil with almost similar levels of oleic (41%) and linoleic acids (43%) and some palmitic (9%) and stearic acids (6%) [21]. Sesame oil can be classified under oleic-linoleic acid group. The palmitic and stearic [22] are dominant saturated acids. The non-saponifiable fraction of the sesame seed oil entails sterols, lignans, sesamins, nitroslactone, and sesamolin. Sesamin and sesamolin are not found in any other vegetable oil. Sesamin is present in the concentrations of 0.5% to 1%. Sesamol, a phenolic antioxidant, is present in traces [23]. 

Castor oil is obtained from *Ricinus communis,* which belongs to the family of Euphorbiaceae*.* Castor oil obtained from the seeds and young leaves has been traditionally used as laxative and purgative. The gas-liquid chromatography of castor oil showed the availability of ester form of palmitic (1.2%), steric (0.7%), arachidic (0.3%), hexadecenoic (0.2%), oleic (3.2%), linoleic (3.4%), linolenic (0.2%), ricinoleic (89.4%), and dihydroxy stearic acids [24]. The chromatography-mass spectrometry (GC-MS) analysis of castor oil demonstrated the presence of alpha thujone (31.71%) 1,8-cineole (30.98%), alpha-pinene (16.88%), camphor (12.92%), and camphene (7.48%) [25]. Lupeol and 30-norlupan-3β-ol-20-one were isolated from the coat of castor bean [26].

With this background in mind, the present study aimed to trace out the constituents present in the soleshine using GC-MS analysis and study the inhibition of these compounds against various drug targets of dermatophytes by molecular docking method. 

## Materials and Methods

***Gas chromatography-mass spectrometry*** 

The phytochemical analysis of the extract of soleshine, a polyherbal formulation, was performed by GC-MS equipment (Thermo Scientific Co., Thermo GCTRACE ultra, version 5.0, Thermo MS DSQ II). The experimental conditions of GC-MS system included TR 5MS capillary standard non-polar column, dimension of 30 Mts, ID of 0.25 mm, and film thickness of 0.25 μm. The flow rate of mobile phase (carrier gas: helium) was set at 1.0 mL/min. In the gas chromatography division, the temperature (oven temperature) was 40°C raised to 250°C at 5°C/min, and injection volume was 1 μl. The samples dissolved in chloroform were run fully at a range of 50650 m/z, and the results were compared by using Wiley Spectral library search program. 

***Preparation of protein*** ***by homology modeling***

The three-dimensional (3D) structures of drug targets selected in this study were not available in the RCSB database. Therefore, their 3D structures were obtained by homology modelling. The primary structures of 1,3-β-glucan synthase (Uniprot accession no: P38631), chitinase (Uniprot accession no: P40954), fungalysin (Uniprot accession no: Q8NIB6), and lumazine synthase (Uniprot accession no: P50861) were obtained in FASTA format from the UniprotKB database. 

The homology protein templates of the 1, 3-β-glucan synthase (template RCSB accession code: FKS1), chitinase (template RCSB accession code: 4TX6), fungalysin (template RCSB accession code: 4K90), and lumazine synthase (template RCSB accession code: 1EJB) were obtained from the RCSB database. The homology modeling was performed using MODELLER software (version: 9.0) using EasyModeller as the graphical user interface. The query sequence and template of these proteins were submitted and processed to generate the 3D structures of the proteins. The generated 3D structure of the macromolecule or model protein were validated by means of Ramachandran Plot and SAVES online server tool.

***Preparation of ligand***

The two compounds, namely 2,6-Octadienal, 3,7-dimethyl and 2-methyl-2-pentene, were selected based on the covered area and height, which were 40.15% and 46.17% in the former compound and 31.90% and 23.33% in the latter one, respectively. The SDF files of these compounds were obtained from Pubchem database. The SDF files were converted into PDB file format using OPEN BABEL software. 

***Protein-ligand docking***

The initial rough docking was performed in iGEMDOCK software (version 2.0) with a population size of 150 and 70 generations, set as default. Protein-ligand docking was carried out in Autodock Vina [27], which is an interactive molecular graphics program for calculating and displaying the feasible docking modes of protein and ligand pairs presented in a hierarchy based on their binding affinities.

***Lead-likeliness properties***

The SWISS ADME, a free web tool was used to generate the physicochemical, medicinal, and druglikeness properties of these two compounds. Lipinski's rule [28, 29] also called the rule of five (RO5) is to evaluate the druglikeness or determine if a chemical compound with a certain pharmacological or biological activity has properties that may be active peroral.

***Toxicity***

The toxicity of the compounds was detected with admetSAR, a free online web server. This server provides the possible toxicity profile of the compounds with the values suggesting the safety. 

## Results

***Gas chromatography-mass spectrometry analysis***

**Figure 1:**
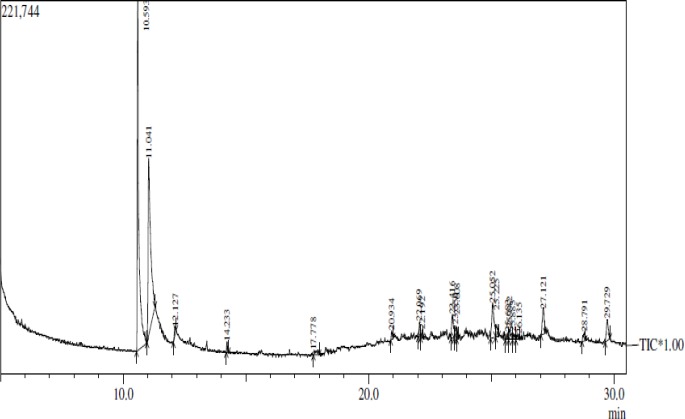
Chromatogram of a polyherbal preparation

Figure 1 depicts the thobtainede chromatogram. The compounds present in soleshine are demonstrated in Table 1. The chromatogram revealed the presence of 20 compounds in the investigated polyherbal preparation. The two compounds, namely 2,6-Octadienal, 3,7-dimethyl and 2-methyl-2-pentene, were selected for further study on the basis of the covered area and height, which were 40.15% and 46.17% in the former compound and 31.90% and 23.33% in the latter one, respectively.

**Table 1:** Compounds obtained from the gas chromatography-mass spectrometry analysis of a poly herbal preparation

**Compound No.**	**R-time**	**Area%**	**Height%**	**Name of the compound**	**Chemical formula**	**Mol. weight**
1	10.593	40.15	46.17	2,6-Octadienal, 3,7-dimethyl	C_10_H_16_O	152
2	11.041	31.90	23.33	2-Pentene, 2-methyl	C_6_H_12_	84
3	12.127	0.85	0.93	1,2,3-Propanetriol, diacetate	C_7_H_12_O_5_	176
4	14.233	0.62	1.4 1	Butane, 1,1'-oxybis[4-chloro	C_8_H_16_ClO	198
5	17.778	1.07	0.62	Oxalic acid, cyclobutyl heptyl ester	C_13_H_22_O_4_	242
6	20.934	0.69	1.13	Docosane	C_22_H_46_	310
7	22.069	1.08	1.92	2-Octyldodecan-1-ol	C_20_H_42_O	298
8	22.192	0.64	1.05	3-Methyl-1-(2-tetrahydropyranyloxy)-2-butene	C_9_H_14_O_3_	170
9	23.416	2.84	3. 12	Dihexylsulfide	C_12_H_26_S	202
10	23.550	1.29	1.65	Sulfurous acid, cyclohexylmethyl heptadecyl ester	C_24_H_48_O_3_S	416
11	23.608	0.59	1.4 1	Beta-l-rhamnopyranoside, phenyl-2,3-o-ethylboranediyl-4-o-benzyl	C_21_H_25_BO_5_	368
12	25.052	5.66	4.60	1-(Hexadecyloxy)ethylene	C_18_H_36_O	268
13	25.225	1.38	1.61	2-Butyn-1-al diethyl acetal	C_8_H_14_O_2_	142
14	25.683	0.71	0.96	5-(Benzyloxy)-7,7-dimethyl-1,3,8-nonatriene	C_18_H_24_O	256
15	25.742	1.51	1.28	1,2,4-Thiadiazol-5(4h)-one, 3-methyl-4-propyl	C_6_H_10_N_2_OS	158
16	25.883	0.85	0.89	2,2-Dimethyl-1-propyl phenyl telluride	C_11_H_14_Te	276
17	26.135	0.66	0.73	1-(2-Propenyl)-1-(tosyloxy)cyclopropane	C1_3_H_16_O_3_S	252
18	27.121	3.56	3.37	Dodecane, 1,1'-oxybis-	C_24_H_50_O	354
19	28.791	1.04	1.15	Di-isodecyl phthalate	C_28_H_46_O_4_	446
20	29.729	2.93	2.68	Spiro[cyclopentane-1,2'(1'h)-quinoxaline], 3'-(4-morpholinyl)-6',8'-dinitro	C_16_H_19_N_5_O_5_	361

R-time: retention time

**Figure 2:**
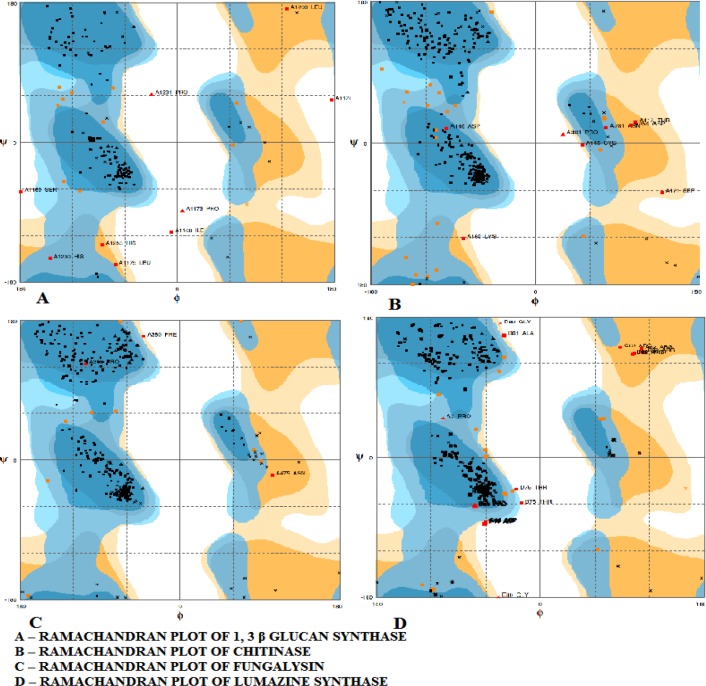
Ramachandran plot of the four proteins showing most of the residues clustered tightly in the most-favoured regions with very few outliers

***Homology modeling***

The 3D structures obtained by homology modeling were validated with the aid of the Ramachandran plot as shown in Figure 2. The Ramachandran plot demonstrated that most of the residues clustered tightly in the most-favoured regions with very few outliers for all the drug targets. The Ramachandran plot (discovered by G. N. Ramachandran, C. Ramakrishnan, and V. Sasisekharan [30]) is a way to visualize the dihedral angles, namely ψ (psi) and φ (phi), of a protein backbone [31]. Given that steric hindrances occur between adjacent atoms within a protein structure, ψ (psi) and φ (phi) values are usually constrained within the specific areas of the plot for ordered structures, such as helices and sheets. The 1,3-β-glucan synthase protein structure contained 87.5% amino acid residues in the favoured region, 6.9% in the allowed region, and 5.6% in the disallowed region. 

In case of chitinase, the favoured, allowed, and disallowed regions were 91.6%, 6.1%, and 2.3%, respectively. The fungalysin was 96.9% favoured, 2.3% allowed, and 0.8% disallowed. Furthermore, lumazine synthase was calculated as 93.8% favoured, 3.6% allowed, and 2.6% disallowed. The proteins were further validated using SAVES server.

***Molecular docking***

The target proteins, namely 1,3-β-glucan synthase, chitinase, fungalysin, and lumazine synthase, were docked with 2,6-octadienal, 3,7-dimethyl and 2-methyl-2-pentene by iGEMDOCK and Autodock Vina. Clotrimazole, which is an anti-fungal drug, was also included in the docking study. The energy values and the binding affinities are presented in Table 2. The energy values obtained by iGEMDOCK of the drug targets of 1,3-β-glucan synthase, chitinase, fungalysin, and lumazine synthase, along with 2,6-octadienal, 3,7-dimethyl, 2-methyl-2-pentene, and clotrimazole were -69.94/-65.71/-128.424, -76.36/-83.77/-143.803, -67.49/-67.88/-105.115, and -66.76/-75.84/-115.185 Kcal/mol, respectively. 

**Figure 3:**
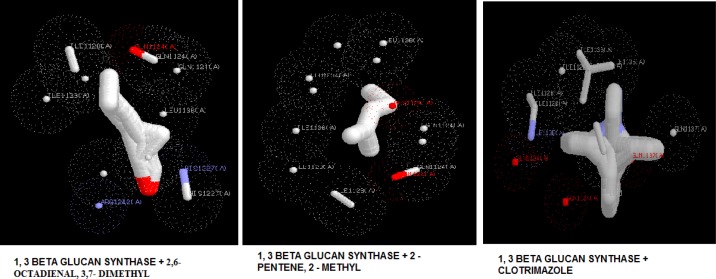
Docking poses of 1,3-β-glucan synthase with 2,6-octadienal, 3,7-dimethyl, 2-methyl-2-pentene, and clotrimazole

**Figure 4:**
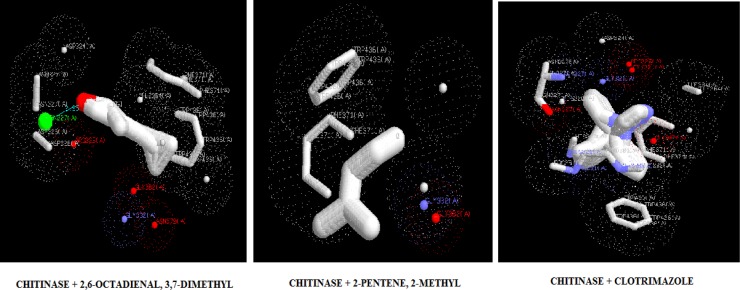
Docking poses of chitinase with 2,6-octadienal, 3,7-dimethyl, 2-methyl-2-pentene, and clotrimazole

**Figure 5:**
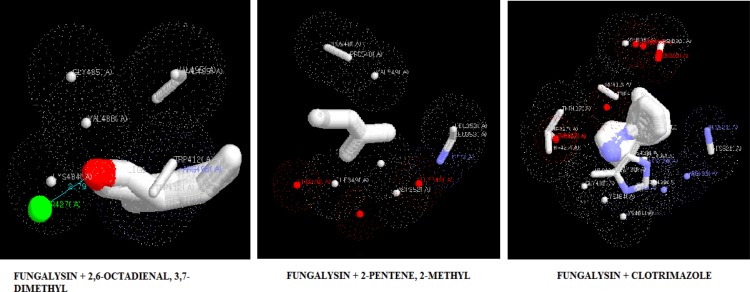
Docking poses of fungalysin with 2,6-octadienal, 3,7-dimethyl, 2-methyl-2-pentene, and clotrimazole

**Figure 6:**
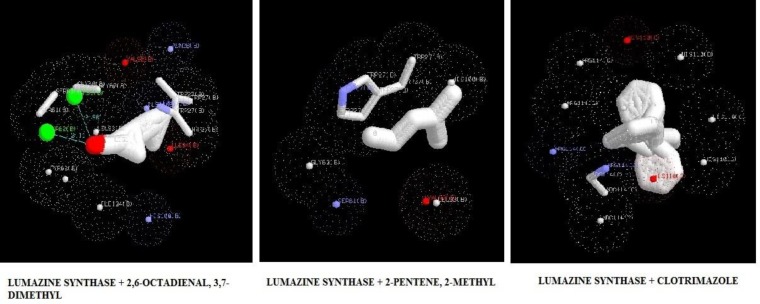
Docking poses of lumazine synthase with 2,6-octadienal, 3,7-dimethyl, 2-methyl-2-pentene, and clotrimazole

The binding affinity values obtained by Autodock Vina for 1,3-β-glucan synthase, chitinase, fungalysin, and lumazine synthase, along with 2,6-octadienal, 3,7-dimethyl, 2-methyl-2-pentene, and clotrimazole were -5.8/-5.8/-7.1, -7.2/-7.2/-10.1, -5.1/-5.1/-7.9, and -5.8/-5.8/-8.1, respectively. The docking pose of the compounds with various drug targets were analyzed with LigPlot^+^ software tool. Figures 3-6 displays the docking poses of various compounds with their protein drug targets. The docking poses were analyzed, and the amino acid residues involved in the various interactions were evaluated

**Table 2:** Results of rough docking and accurate docking performed with a software iGEMDOCK and Autodock Vina between the drug targets with ligands (2,6-octadienal, 3,7-dimethyl and 2-methyl-2-pentene) and clotrimazole

**S no**	**Drug targets or protein with ligand**	**Rough docking energy values with iGEMDOCK**					**Binding affinity with Autodock Vina**		
		**Total energy ** **(Kcal/mol)**	**V.D.W. ** **(Kcal/mol)**	**H. Bond ** **(Kcal/mol)**	**Electrostatic ** **(Kcal/mol)**	**Aver Con pair ** **(Kcal/mol)**	**Binding affinity**	**RMSD/UB**	**RMSD/LB**	
1	1,3-β-Glucan synthase + 2, 6-octadienal, 3,7-dimethyl	-68.81	-62.55	-6.25	0	34.36	-5.8	0	0	
2	1,3-β-Glucan synthase + 2-methyl-2-pentene	-39.50	-39.50	0	0	40.5	-4.2	0	0	
3	1,3-β-Glucan synthase + clotrimazole	-128.424	-124.853	-3.5713	0	29.4	-7.1	0	0	
4	Chitinase + 2, 6-octadienal, 3,7-dimethyl	-80.59	-75.11	-5.47	0	40.72	-7.2	0	0	
5	Chitinase + 2-methyl-2-pentene	-41.99	-41.99	0	0	42.33	-5.4	0	0	
6	Chitinase + clotrimazole	-143.803	-140.303	-3.5	0	38	-10.1	0	0	
7	Fungalysin + 2, 6-octadienal, 3,7-dimethyl	-64.14	-53.91	-10.22	0	33.27	-5.1	0	0	
8	Fungalysin + 2-methyl-2-pentene	-34.21	-34.21	0	0	34.5	-3.7	0	0	
9	Fungalysin + clotrimazole	-105.115	-100.119	-4.99543	0	24.08	-7.9	0	0	
10	Lumazine synthase + 2,6-octadienal, 3,7-dimethyl	-71.77	-71.77	0	0	40.27	-5.8	0	0	
11	Lumazine synthase + 2-methyl-2-pentene	-40.94	-40.94	0	0	39.5	-4.2	0	0	
12	Lumazine synthase + clotrimazole	-115.185	-109.823	-5.36169	0	31.44	-8.1	0	0	

VDW: Van der Waals force, H Bond: hydrogen bond, RMSD: root mean square deviation, UB: upper bound, LB: lower bound

***Druglikeness and other properties***

Table 3 presents the general properties of compounds 2,6-octadienal, 3,7-dimethyl and 2-methyl-2-pentene, such as molecular formula, chemical structure, simplified molecular input line entry specification, and international union of pure and applied chemistry name. Table 4 tabulates the molecular weight, number of atoms, fraction CSP3, number of rotatable bonds, molar refractivity, and topological polar surface area. The molecular weights, number of atoms, molar refractivity, and polar surface area were less than 500, 20, 50, and 20 Å², respectively, in 2,6-octadienal, 3,7-dimethyl and 2-methyl-2-pentene, representing good oral bioavailability.

**Table 3:** General properties of 2,6-octadienal, 3,7-dimethyl and 2-methyl-2-pentene

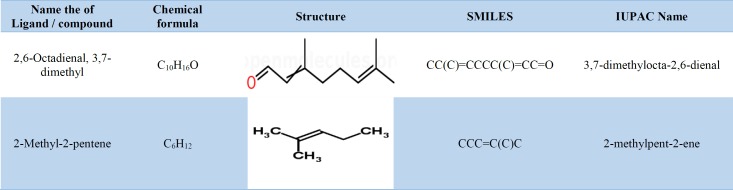

SMILES: Simplified Molecular Input Line Entry Specification, IUPAC: International Union of Pure and Applied Chemistry

**Table 4:** Physicochemical properties of 2,6-octadienal, 3,7-dimethyl and 2-methyl-2-pentene

**Name of ligand**	**Molecular Weight** **(g/mol)**	**Num. heavy atoms**	**Num. arom. heavy atoms**	**Fraction CSP3**	**Num. rotatable bonds**	**Num. H-bond acceptors**	**Num. H-bond donors**	**Molar refractivity**	**TPSA (** ^o^ **A** ^2^ **)**
2,6-Octadienal, 3,7-dimethyl	152.23	11	0	0.5	4	1	0	49.44	17.07
2-Methyl-2-pentene	84.16	6	0	0.67	1	0	0	30.48	0

TPSA: Topological Polar Surface Area

**Table 5:** Lipophilicity and hydrophilicity of 2,6-octadienal, 3,7-dimethyl and 2-methyl-2-pentene

**Name of ** **ligand**	**Lipophilicity**	**Hydrophilicity**								
	**Consensus Log P**_o/w_	**Log S (ESOL)**	**Solubility**	**Class**	**Log S (Ali)**	**Solubility**	**Class**	**Log S (SILICOS-IT)**	**Solubility**	**Class**
2,6-Octadienal, 3,7-dimethyl	2.71	-2.43	5.67E-01	Soluble	-3.05	1.34E-01	Soluble	-1.96	1.66E+00	Soluble
2-Methyl-2-pentene	2.43	-2.01	8.24E-01	Soluble	-2.37	3.56E-01	Soluble	-1.43	3.16E+00	Soluble

o/w: octanol/water

Table 5 demonstrates the octanol-water partition coefficient values of 2,6-octadienal, 3,7-dimethyl and 2-methyl-2-pentene. As indicated in this table, these values were within the permissible range of -0.4 to +5.6, implying a good lipophilic compound. 2,6-Octadienal, 3,7-dimethyl and 2-methyl-2-pentene compounds were mostly soluble in aqueous medium as the log S was less than -4.0. Table 6 illustrates the pharmacokinetic properties of 2,6-octadienal, 3,7-dimethyl and 2-methyl-2-pentene. According to the results, the oral bioavailability was high for 2,6-octadienal, 3,7-dimethyl and low for 2-methyl-2-pentene. Both compounds cross blood brain barrier, and none of them affected the liver cytochrome P450 enzymes; however, penetration through skin was better for both of the compounds.

Based on Table 7, 2, 6-octadienal, 3,7-dimethyl and 2-methyl-2-pentene followed the Lipinski’s rule of 5 [28, 29] and other filters, like Veber [32] and Egan [33], with one violation for Ghose filter [34] of 2,6-octadienal, 3,7-dimethyl, three violations for 2-methyl-2-pentene and two violations of Muegge filter [35]. For a new drug molecule, the bioavailability score [36] is to predict the probability of a new drug that have at least 10% oral bioavailability in rodents. The filters for leadlikeliness, like pains filter [37] and brenk filter [38], were obeyed for 2, 6-octadienal, 3,7-dimethyl and 2-methyl-2-pentene. A new drug compound with a molecular weight of 250-350, XLog P less than 3.5, rotatable bonds of 7, and synthetic accessibility 2.5 can be an Investigational New Drug (IND).

**Table 6:** Pharmacokinetics properties of 2,6-octadienal, 3,7-dimethyl and 2-methyl-2-pentene

**Name of** **ligand**	**GI absorption**	**BBB permeability**	**P-gp substrate**	**CYP 1A2 Inhibitor**	**CYP2C19 Inhibitor**	**CYP2C9 Inhibitor**	**CYP2D6 Inhibitor**	**CYP3A4 Inhibitor**	**Log K**_p_ **(skin permeation) cm/s**
2,6-Octadienal, 3,7-dimethyl	High	Yes	No	No	No	No	No	No	-5.08
2-Methyl-2-pentene	Low	Yes	No	No	No	No	No	No	-4.88

GI absorption: Gastrointestinal absorption, BBB: blood brain barrier, CYP: cytochrome P

**Table 7:** Druglikeness and leadlikeness of 2,6-octadienal, 3,7-dimethyl and 2-methyl-2-pentene

**Name of ** **ligand**	**Druglikness**						**Leadlikeness**			
	**Lipinski**	**Ghose**	**Veber**	**Egan**	**Muegge**	**Bioavailability score**	**Pains**	**Brenk**	**Leadlikeness**	**Synthetic accessibility**
2,6-Octadienal, 3,7-dimethyl	0	1	0	0	2	0.55	0	3	1	2.49
2-Methyl-2-pentene	0	3	0	0	2	0.55	0	1	1	2.08

**Table 8:** Toxicity profile of 2,6-octadienal, 3,7-dimethyl and 2-methyl-2-pentene

**Name of ligand**	***hERG*** ** inhibition**	**AMES toxicity**	**Carcinogenicity** **(Class III)**	**Acute oral toxicity**	**Rat acute toxicity (LD 50 mg/)**
2,6-Octadienal, 3, 7-dimethyl	0.9220	0.9133	0.5545	0.8232	1.6001
2-Methyl-2-pentene	0.9451	0.9354	0.5328	0.7693	1.6545

* hERG*: human Ether-a-go-go related gene

Table 8 tabulates the toxicity profile of the compounds of 2,6-octadienal, 3,7-dimethyl and 2-methyl-2-pentene, which were non-toxic in hERG, AMES toxicity, acute oral toxicity, and LD50 in rats. Regarding the carcinogenicity, 2,6-octadienal, 3,7-dimethyl was found to be non-carcinogenic, and 2-methyl-2-pentene was revealed to be an alarming sign as the median toxic dose was above 10 mg per kg body weight per day. 

## Discussion

The GC-MS analysis revealed the presence of many compounds in the polyherbal preparation under investigation. The first major compound, namely 2,6-octadienal, 3,7-dimethyl, showed the retention time, area, and height of 10.593%, 40.15%, and 46.17%, respectively. Regarding the other major compound (i.e., 2-methyl-2-pentene), the retention time, area, and height were 11.041%, 31.90%, and 23.33%, respectively. These two compounds showed good binding affinity with the biological targets of fungal organism, good total energy, and Van der Waals force as depicted in Table 2. 

The two compounds present in the polyherbal preparation had a good investigational new drugs as they followed Lipinski‘s rule of five, and also obeyed druglikeness and leadlikeness properties, as given in tables 5-8. Many studies have been conducted to identify the constituents of the plant extracts having antifungal activities. According to Jeyam et al., 20 phytochemical constituents interacted with 1,3-β-glucan synthase. They showed that the inhibition of 1,3-β-glucan synthase was better by the Echinocandin group of antifungal agents [39]. 

In another similar study, Mahmoud found that the organic extracts of neem leaves demonstrated more antifungal activity than its aqueous extract [40]. In a study performed by Kannahi, the ethanolic extract of *Lawsonia inermis* showed 100% antifungal activity; however, its aqueous extract demonstrated no activity [41]. According to Laszlo Sami, the inhibition of chitinase by allosamindin showed the growth and survival of fungal organism [42]. In this study, an attempt was made to identify the bioactive compounds of a polyherbal preparation by in silico methods. According to the results, the two compounds showed a good antifungal activity as they inhibited the enzymes responsible for the survival of fungal organism; furthermore, they were appropriate for the lead molecules. 

## Conclusion

The polyherbal preparation should be further explored to prepare investigational new drugs for the treatment of dermatophytosis. The compounds present in this preparation could be a good target for the proteins that may hamper the survival and growth of the fungi, including dermatophytes.
