# Erratum for Nagesh et al., phytochemical analysis and docking study of compounds present in a polyherbal preparation used in the treatment of dermatophytosis 

**DOI:** 10.18502/cmm.4.1.33

**Published:** 2018-03

**Authors:** Simhadri VSDNA Nagesh, Muthuchamy Muniappan, Iyanar Kannan, Subramanyam Viswanathan

**Affiliations:** 1 Department of pharmacology Bharath University, Chennai , India; 2 Department of Pharmacology, Sree Balaji Medical College and Hospital, Chennai, India; 3 Department of Microbiology, Tagore Medical College and Hospital, Chennai, India; 4 Department of Pharmacology, Meenakshi Medical College and Hospital, Chennai, India

**ERRATUM**

Volume 3, no. 4, 6-14, 2017, DOI: 10.29252/cmm.3.4.6[1]. This erratum adds the primary affiliation of Simhadri VSDNA Nagesh, a Research scholar. The affiliation line should appear as shown above.
